# Draft genome sequence of *Wickerhamomyces anomalus* LBCM1105, isolated from cachaça fermentation

**DOI:** 10.1590/1678-4685-GMB-2019-0122

**Published:** 2020-06-08

**Authors:** Aureliano C. Cunha, Renato A. Corrêa dos Santos, Diego M. Riaño-Pachon, Fábio M. Squina, Juliana V. C. Oliveira, Gustavo H. Goldman, Aline T. Souza, Lorena S. Gomes, Fernanda Godoy-Santos, Janaina A. Teixeira, Fábio Faria-Oliveira, Izinara C. Rosse, Ieso M. Castro, Cândida Lucas, Rogelio L. Brandão

**Affiliations:** 1 Universidade Federal de Ouro Preto, Laboratório de Biologia Molecular e Celular, MG, Brazil.; 2 Centro Nacional de Pesquisa em Energia e Materiais (CNPEM), Laboratório Nacional de Ciência e Tecnologia do Bioetanol (CTBE), Campinas, SP, Brazil.; 3 Universidade de São Paulo, Centro de Energia Nuclear na Agricultura, Laboratório de Biologia Computacional, Evolutiva e de Sistemas, Piracicaba, SP, Brazil.; 4 Universidade de Sorocaba, Programa de Pós-Graduação em Processos Tecnológicos e Ambientais, Sorocaba, SP, Brazil.; 5 Universidade de São Paulo, Faculdade de Ciências Farmacêuticas, Ribeirão Preto, SP, Brazil.; 6 Universidade do Minho, Centro de Pesquisa Molecular e Ambiental (CBMA), Instituto de Ciência e Inovação para a Bio-Sustentabilidade (IB - S), Braga, Portugal.

**Keywords:** Non-conventional yeast, glycerol, “de novo” assembly, glycerol

## Abstract

*Wickerhamomyces anomalus* LBCM1105 is a yeast isolated from *cachaça* distillery fermentation vats, notable for exceptional glycerol consumption ability. We report its draft genome with 20.5x in-depth coverage and around 90% extension and completeness. It harbors the sequences of proteins involved in glycerol transport and metabolism.

*Wickerhamomyces anomalus* (synonyms *Pichia anomala, Hansenula anomala* and *Candida pelliculosa*) are found in several diverse natural habitats, frequently associated with spoilage or processing of food and grain products (Passoth *et al.*, 2006). Different strains of *W. anomalus* were reported (i) to be able to grow on a wide variety of conditions, including different carbon and nitrogen sources (Conceição *et al.*, 2015; Cunha *et al.*, 2019), at both low and high pH (2.0 to 12.4) and from 3 to 37 °C (Fredlund *et al.*, 2002), (ii) to be highly tolerant to different stress conditions, like osmotic stress (salt), high concentrations of ethanol, and the presence of heavy metals, and (iii) to produce ethanol from glucose, sucrose or xylose. *W. anomalus* strains have also been reported to display constitutive cyanide-resistant alternative oxidase (Cunha *et al.*, 2019). *W. anomalus* has been used as a cell factory for the production, among others, of enzymes (Díaz-Rincón *et al.*, 2017), biosurfactants (Teixeira Souza *et al.*, 2018) and fermented-beverages (Aplin *et al.*, 2019). Although *W. anomalus* strains show a high industrial versatility, only two strains have its genome sequenced to date (Schneider *et al.*, 2012; Riley *et al.*, 2016).

*W. anomalus* strain LBCM1105 (previously LBCM105) was isolated from sugarcane fermentation vats in a cachaça distillery in Brazil (Conceição *et al.*, 2015), (S22.099694, W41.511090). Extraction of DNA was carried out using the phenol/chloroform method, and purification was performed using the PowerClean DNA Clean-UP kit (MoBio, QIAGEN, Carlsbad, US). The genome size was determined by flow cytometry as previously described (Hare and Johnston, 2011). Cell samples were stained with 2 μM Sytox Green (Thermo Fisher Scientific, MA, US) and the assessment was made in triplicate. The genomic library for sequencing was prepared with the Nextera DNA Library kit (Illumina, San Diego, California, US). Genome sequencing (1.0 million paired-end reads of 151 bp) was performed with an Illumina HiSeq 2500. Quality trimming, and the removal of reads shorter than 90 nucleotides, were carried out using Trimommatic v.0.32 (Bolger *et al.*, 2014). The genome was assembled into contigs (20.5 x in depth coverage, ≥ 1 kb) using SPAdes v.3.11.1, dipSPAdes mode (Bankevich *et al.*, 2012). The completeness was evaluated by BUSCO v.3.0 (Simão *et al.*, 2015), using the Fungi and Saccharomycetales datasets. Genome statistics were computed with QUAST v5.0.2 (Gurevich *et al.*, 2013). A multilocus phylogenetic analysis was performed using RAxML v.8 (Stamatakis, 2014) building a Maximum Likelihood tree based on DNA sequences from the Internal Transcribed Spacers 1 and 2 (ITS1, ITS2), the large and small ribosomal subunits (LSU, SSU), and the Elongation Factor-1α (EF-1α) from species within the genus *Barnettozyma*, *Wickerhamomyces* and *Candida*. The species and the accession numbers of loci LSU, SSU and EF-1α of the related microorganism were previously described (Kobayashi *et al.*, 2017). The accession numbers for ITS are listed in Figure S1). *Saccharomyces cerevisiae* S288c was used as the outgroup. The sequences of the loci SSU, LSU and EF-1α of the LBCM1105 strain were identified *via* Blast searches using the proper sequences from *W. anomalus* NRRL Y-366 as baits (SSU- EF550479.1, LSU- EF550341.1 and EF-1α- EF552565.1). ITS1 and ITS2 sequences from *W. anomalus* LBCM1105 was extracted using ITSx v.1.0.11 (Bengtsson-Palme *et al.*, 2013). The sequences of ITS1, ITS2, LSU and SSU were aligned using MXSCARNA v.2.1 (Tabei *et al.*, 2008), and of EF-1α protein using MAFFT v.7 (Katoh *et al.*, 2017). rtREV was selected using IQ-TREE v1.6 (Nguyen *et al.*, 2015) as the best evolutionary model for the EF-1α phylogenetic analysis. All the alignments were concatenated in a supermatrix using FASconCAT v.1.04 (Kuck and Meusemann, 2010), which was used to conduct a partitioned phylogenetic analysis. A phylogenetic tree based on the alignments and in the evolutionary model (rtREV for EF-1α and GTR for the others – ITS1, ITS2, LSU and SSU), was inferred using RAxML v.8.4 (Stamatakis, 2014), with 1,000 bootstrap replicates. Genome annotation was done using Augustus v3.3.1 (Stanke *et al.*, 2008) and BRAKER2 v2.1.2 (Hoff *et al.*, 2019), using as extrinsic evidence for training the proteins of *W. anomalus* deposited in GenBank. Proteins related to glycerol transport and metabolism were identified in the LBCM1105 genome using Blastx.

The GC content of the genome was 34.51%. The phylogenetic analysis (Figure S1) confirmed that LBCM1105 is, in fact, a strain within *W. anomalus*, in the same clade with the *W. anomalus* NRRL Y-366-8, with a bootstrap of 100%. Moreover, according to flow cytometry analyses, the genome of strain LBCM1105 is 13.93 ± 0.11 Mb. The total genome assembly corresponds to 12.72 Mb, *i.e*., 91.31% of the expected size, and 89.89% in relation to the genome of the *W. anomalus* strain NRRL Y-366-8 (GCA_001661255.1) which has a genome size of 14.15 Mb. The completeness of the genome assembly, as evaluated on the gene space by BUSCO, was 88.6% for the fungi dataset (290 genes) and 85.5% for the Saccharomycetales dataset (1711 genes). Half of the data is present in 51 scaffolds (L50) larger than 76 kb (N50), the largest being 229 kb. The total number of contigs was 389 with 6,812 predicted protein-coding genes. This number is similar to the 6,421 ORFs previously reported from the genome of *W. anomalus* NRRL Y-366-8 (Riley *et al.*, 2016), and to the 5,885 ORFs of *Saccharomyces cerevisiae* (Goffeau *et al.*, 1996). We compared the genome annotation of LBCM1105 (Augustus and BRAKER2) to that of NRRL Y-366-8, *S. cerevisiae* S288c and *W. ciferrii* using OrthoFinder (Emms and Kelly, 2015). This comparison clearly showed that most predicted genes in LBCM1105 can be assigned to orthologous groups and are shared with the other genomes in the analysis (Figure S2 and Table 1). This Whole Genome Shotgun project has been deposited at DDBJ/ENA/GenBank under the accession SHLV00000000. The version described in this paper is version SHLV01000000.

**Table 1:** Comparison of groups of orthologous genes between *W. anomalus* LBCM1105 with two annotation strategies A) Augustus, B) BRAKER2, *W. anomalus* NRRL Y-366-8, *W. ciferrii* NRRL Y-1031 and *S. cerevisiae* S288c.

Groups of orthologous genes	LBCM1105-A	LBCM1105-B	S288c	NRRL Y-366-8	NRRL Y-1031
Number of genes in strains/species	6812	6159	6002	6421	6702
Number of genes in orthogroups	5965	6106	4651	6227	5936
Number of unassigned genes	847	53	1351	194	766
Percentage of genes in orthogroups	87,6	99,1	77,5	97,0	88,6
Number of species-specific orthogroups	0	0	7	0	7
Number of genes in species-specific orthogroups	0	0	17	0	79

DNA sequences from *S. cerevisiae* S288c encoding the proteins that perform glycerol transport (the channel Fps1p and the high affinity transporter Stl1p) and metabolism (the consumption Gut1p/Gut2p, the production Gpd1p/Gpd2p and Gpp1p/Gpp2p, as well as the putative pathway Gcy1p, Ypr1p and Dak1p/Dak2p) (Figure 1, and Table 2) were obtained from SGD (https://www.yeastgenome.org) and used to identify the correspondent putative ORFs in the *W. anomalus* LBCM1105 genome. Homologous sequences to the proteins were found (Table 2), in some cases different *S. cerevisiae* proteins aligned to the same protein in the *W. anomalus* LBCM1105 genome, it is not clear which will be the exact function of the LBCM1105's protein, more studies are need to elucidate this. The *W. anomalus* Stl1p was previously studied in detail, showing very high affinity for glycerol (Cunha *et al.*, 2019). The genome sequence presented here provides evidence for the existence of the genes needed to ensure the two glycerol consumption and production pathways known in *S. cerevisiae*. Further studies are required to verify how intrinsic characteristics of these proteins and their expression and regulation are the cause underlying the LBCM1105's extraordinary ability to grow on glycerol as single a carbon source (Conceição *et al.*, 2015).

**Figure 1:**
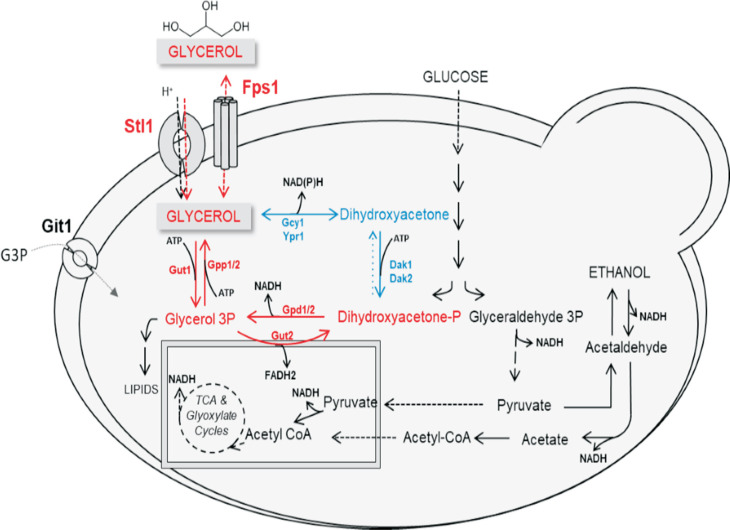
Global yeast metabolism overview focusing on glycerol transport, consumption and production pathways. Red: main metabolic pathway. Blue: alternative pathway with unclear physiological relevance in *S. cerevisiae*.

**Table 2:** Similarity between the *S. cerevisiae* genes encoding the proteins responsible for glycerol transport and metabolism as in Figure 1, and the corresponding sequences identified in the genome of *W. anomalus* LBCM1105. Protein Sequences are available at https://doi.org/10.6084/m9.figshare.11441061.v1

	Protein role		*S. cerevisiae -* SGD database		Gene	Percentage target aligned	Similarity
			Gene	ID			
Regular pathway	Transport	Glycerol channel	*FPS1*	S000003966	g1373.t1	45.3	56%
		Glycerol active permease/ H^+^ symporter	*STL1*	S000002944	g4293.t1	85.4	57%
	Consumption	Glycerol kinase	*GUT1*	S000001024	g1371.t1	91.2	72%
		Glycerol 3P	*GUT2*	S000001417	g5045.t1	98.8	72%
		dehydrogenase/mitochondria					
	Production	Glycerol 3P dehydrogenase	*GPD1*	S000002180	g1302.t1	100	78%
		Glycerol 3P dehydrogenase	*GPD2*	S000005420	g1302.t1	81.1	82%
		Glycerol 3P phosphatase	*GPP1*	S000002180	g4575.t1	99.2	71%
		Glycerol 3P phosphatase	*GPP2*	S000005420	g4575.t1	99.2	71%
Alternative pathway	Consumption/Production	Glycerol dehydrogenase	*GCY1*	S000005646	g1045.t1	98.7	79%
		Glycerol dehydrogenase	*YPR1*	S000002776	g1045.t1	98.7	78%
	Consumption	Dihydroxyacetone kinase	*DAK1*	S000004535	g4297.t1	98.5	56%
		Dihydroxyacetone kinase	*DAK2*	S000001841	g4297.t1	97.8	52%

## Supplementary Material

The following online material is available for this study

Figure S1 Maximum Likelihood (ML) phylogenetic tree based on DNA sequences from large ribosomal subunit (LSU), small ribosomal subunit (SSU) and Elongation Factor-1α (EF-1α).

Figure S2 Venn Diagram of Groups of Orthologous Genes between *W. anomalus* LBCM1105 (LBCM1105-A: Augustus, LBCM1105-B: BRAKER2), *W. anomalus* NRRL Y-366-8, *W. ciferrii* NRRL Y-1031 and S. cerevisiae S288c.
