# Supplementary material for: Draft genome sequence of Wickerhamomyces anomalus LBCM1105, isolated from cachaça fermentation
Source: Genet Mol Biol. 2020 Jun 8;43(3):e20190122. doi: 10.1590/1678-4685-GMB-2019-0122 (PMC7278976; doi:10.1590/1678-4685-GMB-2019-0122)
Supplement: Supplementary file 1 [file 1415-4757-GMB-43-3-e20190122-suppl1.pdf]

Genetics and Molecular Biology

https://doi.org/10.1590/1678-4685-GMB-2019-0122

# Supplementary material to “Draft genome sequence of *Wickerhamomyces anomalus* LBCM1105, isolated from cachaça fermentation”

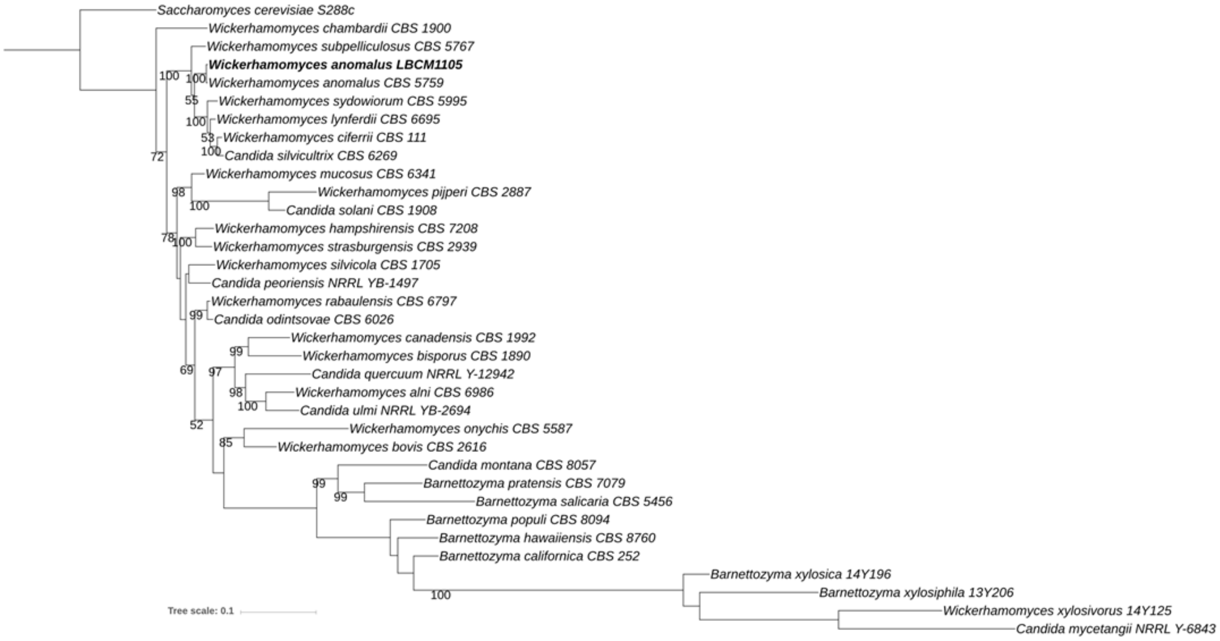


**Figure S1** - Maximum Likelihood (ML) phylogenetic tree based on DNA sequences from large ribosomal subunit (LSU), small ribosomal subunit (SSU) and Elongation Factor-1α (EF-1α). The tree shows the placement of the strain LBCM1105 among strains representative of related species of *Barnettozyma*, *Wickerhamomyces* and *Candida*. The accession numbers of LSU, SSU and EF-1α were described previously (Kobayashi, et al., 2017, doi.org/10.1099/ijsem.0.002233). ITS1 and ITS2 accession number are listed below. *Saccharomyces cerevisiae* (strain S288c) was used as the outgroup in order to root the phylogenetic tree. Bootstrap values (percentages) over 50% from 1,000 bootstrap replicates in ML tree are shown on branches. Accession numbers for the ITS sequences: *B. californica* - NR_138212, *B. hawaiiensis* - NR_153631, *B. populi* - NR_153632, *B. pratensis* - NR_153633, *B. xylosica* - NR_154882, *B. xylosiphila* - LC012021, *C. montana* - NR_153644, *C. mycetangii* - NR_152475, *C. odintsovae* - NR_077084, *C. peoriensis* - NR_154960, C. quercuum - KX525668, *C. silvicultrix* - HM156509, *C. solani* - HM156501, *W. alni* - NR_154966, *W. anomalus* CBS 5759 - NR_111210, *W. bisporus* - KY105897, *W. canadensis* - KY105899, *W. chambardii* - NR_154969, *W. ciferrii* - HM156508, *W. hampshirensis* - HM156504, *W. lynferdii* - HM156505, *W. mucosus* - NR_154970, *W. onychis* - KY105910, *W. pijperi* - HM156502, *W. rabaulensis* - NR_138207, *W. silvicola* - NR_1550121, *W. strasburgensis* - NR_154973, *W. subpelliculosus* - KY105921, *W. sydowiorum* - HM156507, *W. xylosivorus* - LC278379.
