# Supplementary material for: Draft genome sequence of Wickerhamomyces anomalus LBCM1105, isolated from cachaça fermentation
Source: Genet Mol Biol. 2020 Jun 8;43(3):e20190122. doi: 10.1590/1678-4685-GMB-2019-0122 (PMC7278976; doi:10.1590/1678-4685-GMB-2019-0122)
Supplement: Supplementary file 2 [file 1415-4757-GMB-43-3-e20190122-suppl2.pdf]

Genetics and Molecular Biology
https://doi.org/10.1590/1678-4685-GMB-2019-0122

Supplementary material to "Draft genome sequence of *Wickerhamomyces anomalus* LBCM1105, isolated from cachaça fermentation"

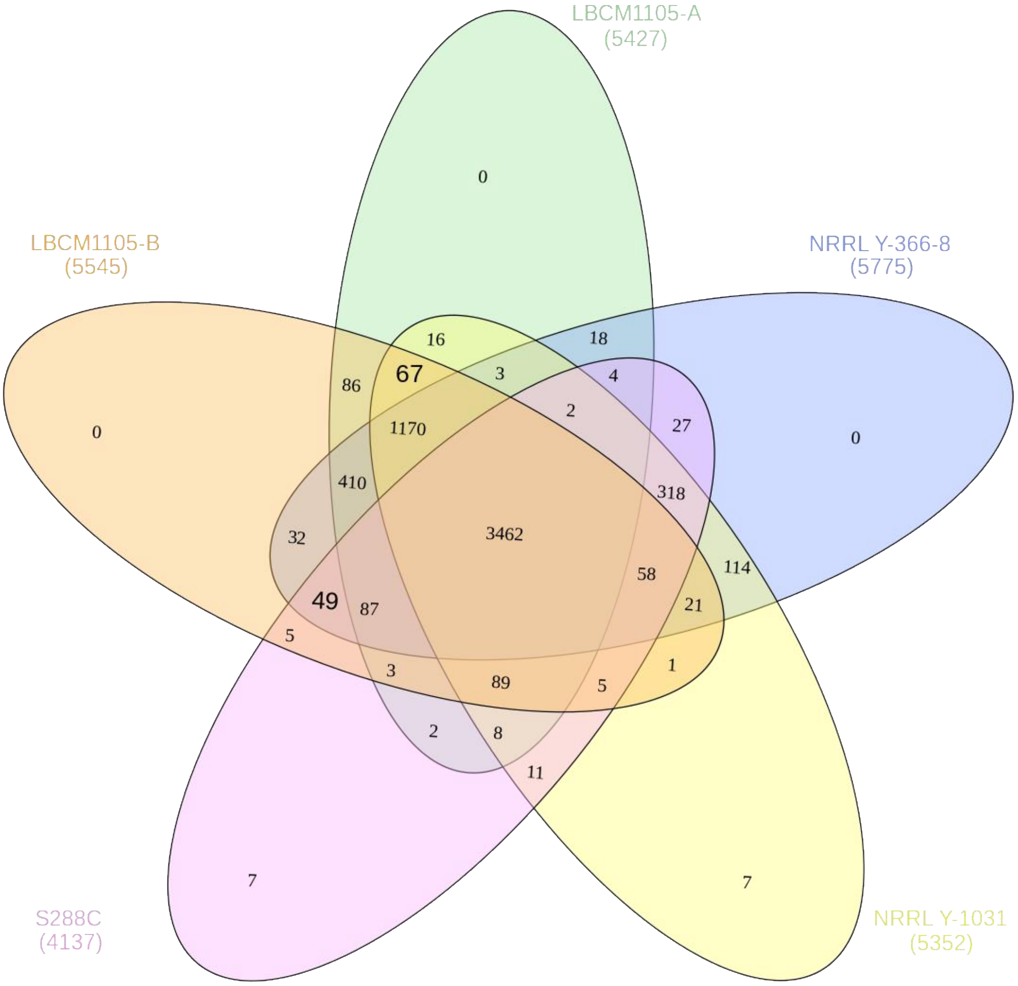

Figure S2 - Venn Diagram of Groups of Orthologous Genes between *W. anomalus* LBCM1105 (LBCM1105-A: Augustus, LBCM1105-B: BRAKER2), *W. anomalus* NRRL Y-366-8, *W. ciferrii* NRRL Y-1031 and *S. cerevisiae* S288c.
